# Supplementary material for: Towards a great ape dictionary: Inexperienced humans understand common nonhuman ape gestures
Source: PLoS Biol. 2023 Jan 24;21(1):e3001939. doi: 10.1371/journal.pbio.3001939 (PMC9873169; doi:10.1371/journal.pbio.3001939)
Supplement: S1 Table — (DOCX) [file pbio.3001939.s001.docx]

**S1 Table.**

Gesture types used, with the meanings for which they are used in both bonobos and chimpanzees. Note that some gestures’ meanings are shared across species, whereas others vary in their Primary and/or Secondary meaning. NA = Not Applicable: there is no described alternate meaning for this gesture in this species.

| *Gesture Type* | *Species* | *Primary Meaning* | *Proportion participants who selected primary meaning (binomial; chance=0.25)* | *Alternate Meaning* | *Proportion participants who selected alternate meaning (binomial; chance = 0.33)* |
| --- | --- | --- | --- | --- | --- |
| Arm Raise | Bonobo | Groom me | 0.40 (n=2362, p<0.001) | Carry me | 0.38 (n=446, p=0.001) |
|  | Chimp | Give me that food |  | NA |  |
| Big Loud Scratch | Bonobo | Groom me | 0.77 (n=4529, p<0.001) | NA | NA |
|  | Chimp | Groom me |  | NA |  |
| Directed Push | Bonobo | Climb on my back | 0.64 (n=3798, p<0.001) | NA | NA |
|  | Chimp | Move into a new position |  | NA |  |
| Mouth Stroke | Bonobo | Give me that food | 0.79 (n=4684, p<0.001) | NA | NA |
|  | Chimp | Give me that food |  | NA |  |
| Object Shake | Bonobo | Let's have sex | 0.22 (n=1312, p=1) | Groom me | 0.22 (n=1297, p=1) |
|  | Chimp | Let's have sex |  | Move away from me |  |
| Present Climb On | Bonobo | Climb on my back | 0.45 (n=2689, p<0.001) | NA | NA |
|  | Chimp | Climb on my back |  | NA |  |
| Present Genitals | Bonobo | Let's have sex | 0.46 (n=2742, p<0.001) | Let’s be friendly | 0.40 (n=2089, p<0.001) |
|  | Chimp | Let's have sex |  | Let’s be friendly |  |
| Present Grooming | Bonobo | Groom me | 0.43 (n=2542, p<0.001) | NA | NA |
|  | Bonobo | Groom me |  | NA |  |
| Reach | Bonobo | Climb on my back | 0.42 (n=2463, p<0.001) | NA | 0.29 (n=448, p=1) |
|  | Chimp | Give me that food |  | Let’s be friendly |  |
| Touch | Bonobo | Climb on my back | 0.62 (n=3649, p<0.001) | NA | 0.31 (n=417, p=1) |
|  | Chimp | Give me that food |  | Let’s be friendly |  |
