# Supplementary material for: Towards a great ape dictionary: Inexperienced humans understand common nonhuman ape gestures
Source: PLoS Biol. 2023 Jan 24;21(1):e3001939. doi: 10.1371/journal.pbio.3001939 (PMC9873169; doi:10.1371/journal.pbio.3001939)
Supplement: S1 Fig — (DOCX) [file pbio.3001939.s002.docx]

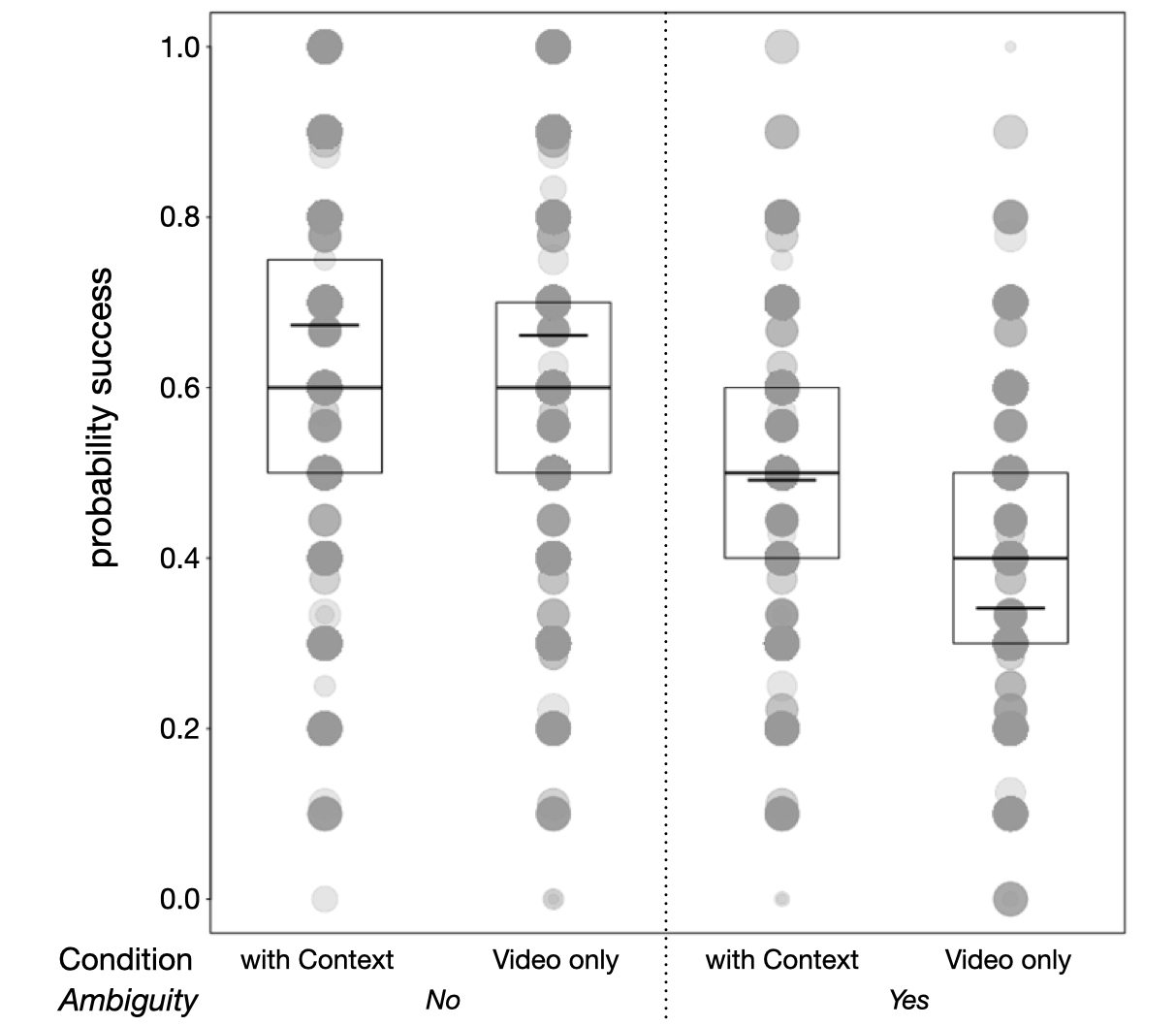


**S1 Fig**. Probability of success, separately for each combination of Context and Ambiguity. Dots show success probabilities per subject, whereby the area of the dots is proportionate to the number of trials per individual and combination of the two factors (range: 1 to 10). Darker dots denote tied observations. The inclusion of brief written information on Context (as opposed to seeing the Video only) provided an at best marginal improvement in participants’ success, most clearly seen when gesture meaning was classed as Ambiguous. The data underlying this Figure can be found at https://doi.org/10.5281/zenodo.7347608
