# Supplementary material for: Towards a great ape dictionary: Inexperienced humans understand common nonhuman ape gestures
Source: PLoS Biol. 2023 Jan 24;21(1):e3001939. doi: 10.1371/journal.pbio.3001939 (PMC9873169; doi:10.1371/journal.pbio.3001939)
Supplement: Attachment — Submitted filename: Graham and Hobaiter-Great Ape Dictionary-PLOS ONE.pdf [file pbio.3001939.s003.pdf]

Referee report on

A great ape dictionary: language-using naïve humans understand non-human ape gestures

August 11, 2022

Recommendation: accept with major revisions

Disclaimer: I have assessed this paper from the perspective of gesture research. I have not sought to assess the strength of the methods or the statistical analysis, concentrating instead on the interpretation of the results.

RECOMMENDATION

This is a fascinating study in a fascinating research program, and the results ought to be eventually published. However the interpretation of the results should be *far* more cautious, and some clarifications should be made at various junctures.

Context and contribution

Hobaiter and Byrne's group have established the existence of rich sets of communicative gestures that are shared among the great apes. In a pilot study in the same group, Kersken et al. found many of these communicative gestures in human infants (in Germany and Uganda). The main question was thus whether human adults do understand ape gestures, especially gestures shared among chimpanzees and bonobos, which might be expected to be shared with humans as well. The present contribution studies precisely this on the basis of 10 chimpanzee/bonobo gestures, and a large-scale online game in which participants are asked to guess the meaning of the gestures.

<u>Main points</u>

Interpretation of the results

The authors write the following in the abstract and in the body of the paper:

Abstract: "We show that humans **retain** an understanding of ape gestural communication, across gesture types and gesture meanings, with information on communicative context providing only a marginal improvement in success. By assessing comprehension, rather than production, we accessed the great ape gestural repertoire for the first time in adult humans. **Cognitive access to an ancestral system of gesture appears to have been retained** after our divergence from other apes, drawing deep evolutionary continuity between their communication and our own."

Text: "Participants were substantially above chance at assigning the "correct" meanings to chimpanzee and bonobo gestures across types, **suggesting that humans have retained their understanding of core features of a gestural system present in our last common ancestor with the Pan genus 6-7 million years ago** (Young et al. 2015)."

In my view, the boldfaced parts are not justified. The authors show that adult humans have an ability to guess the correct use of some ape gestures. This does not tell us whether they do this (i) by way of general intelligence, which might allow them to guess just as well the use of communicative gestures in fish or birds, or (ii) by way of a dedicated cognitive system that has been retained since our last common ancestor with the Pan genus.

For lack of a control (involving for instance bird or fish gestures), I find it impossible to adjudicate between (i) and (ii). Furthermore, when looking at the authors' online game, several things seem striking to me:

1. Introspectively, one has a clear sense of guessing, and of NOT understanding the meaning of the gestures. Of course this could conceal unconscious knowledge of a preserved cognitive system. But an alternative is that one is doing one's best with one's general intelligence.

In this respect, it might be interesting to comment on the subjective confidence that subjects assigned to their judgments, although here too the data would need to be compared to a control of some sort (e.g. one's subjective confidence in understanding human gestures.) Did the subjects get an impression that they were guessing, or did they think they understood a communicative gesture?

2. There are cases in which one can guess the meaning of gestures based on general intelligence alone. Examples:

-One of the video+drawings figures genitals prominently. This makes a 'let's have sex' meaning plausible.

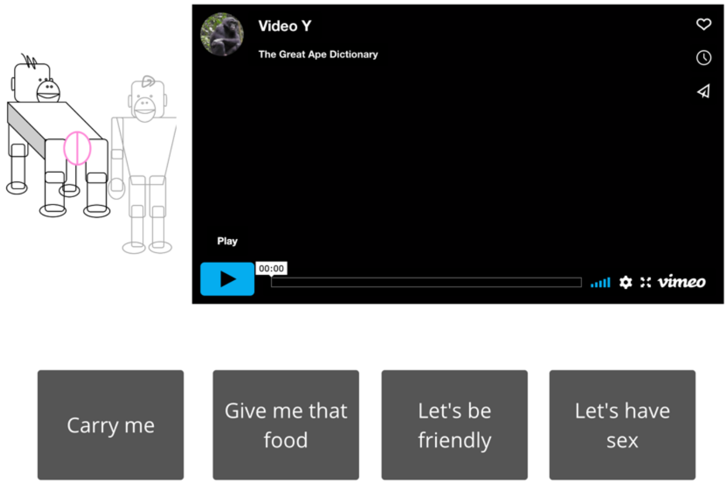

-Similarly, the video of the mouth stroke seems to involve an ape chewing, which makes the correct meaning ('give me that food') particularly plausible.

-Some videos involve iconic gestures, such as some of the grooming gestures.

As things stand, the authors show that there is some understanding of ape gestures in human adults; they do not show that this is because of a preserved cognitive system rather than because of general intelligence.

Selection of the target gestures

1. More should be said about the *criteria* by which the gestures were selected. The article says:

"We selected 10 gesture types for which we were previously able to confirm meaning in both chimpanzees and bonobos (Graham et al. 2018)."

The worry is this: In principle, one would like to test a *random* sample of the gestures to avoid a selection bias. If the researchers selected those gestures that they thought are most transparent to humans, the result would just be that adult humans understand SOME (appropriately selected!) ape gestures. If the selection is random, one can hopefully infer that adult humans GENERALLY understand ape gestures. There is a sizable difference between the two conclusions, but it's hard to tell from the paper which situation we are in.

2. A more minor question is this: in principle, one would expect that those gestures that are found with a constant meaning in gorillas, chimpanzees and bonobos are found with the same meanings in humans as well. This is so because gestures that are shared among the three species should presumably be found in their most common ancestor and could presumably be preserved in humans. Gestures that are *not* shared between bonobos/chimpanzees and gorillas *might* be an innovation (whether in form or in meaning) of the bonobo/chimpanzee lineage, and not shared with humans. I assume it is for practical reasons that the authors focused on bonobo/chimpanzee gestures, but here too additional explanations would help.

3. Similarly, it might be worth explaining in greater detail what is expected in humans when bonobos and chimpanzees assign a different meaning to the same gesture, as is the case for 'directed push' below, from the Supplementary Materials.

Directed Push	Bonobo	Climb on my back	0.64 (n=3798, p<0.001)	NA	NA
	Chimp	Move into a new position		NA	

Title

The title of the contribution isn't optimal. First, it's a stretch to call a 10-gesture list of 'dictionary'. Second, 'language-using' is almost pleonastic when applied to humans, and it doesn't bring any useful information. Third, and less importantly, I find the generic claim a bit overblown ('understand non-human ape gestures'): true, to some extent, on an existential reading ('some non-human ape gestures'); whether it's true more generally (on a generic readings) remains to be seen (this depends in part on clarifications requested above about principles of gesture selection).

'Meaning'

The term 'meaning' is used in the paper for 'Apparently Satisfactory Outcomes' (ASOs). This should be clarified in the paper. To anyone familiar with linguistics, it seems entirely implausible that ASOs are meanings, i.e. the cognitive representations of the informational content of gestures. Rather, ASOs are convenient ways to operationalize the various *uses* of gestures, but it seems clear that the core meanings of these gestures haven't been discovered yet. This is not a problem for the present study, but some readers will be misled if the authors do not state clearly what they understand by 'meaning' (namely ASOs).

Details

–It is fantastic that the stimuli are open access. It would be even better if the relevant gesture drawings and videos were linked to the relevant parts of the paper, especially around Figure 1. This way readers could see exactly what subjects had to assess.

–I thought several of the videos were very hard to decipher, in which case the drawings played an essential role. It might be worth commenting on the fact that subjects saw a *combination* of drawings and videos. In particular, the drawings clearly tell the viewers which part to focus on (as it appears in red).

–When using https://app.gorilla.sc/openmaterials/344409, I got an error message when clicking on:
Great ape dictionary: Error creating schedules

–In the version of the experiment for Kids under 12, subjects are told the right answer after they have submitted theirs. But some gesture videos appear twice (example below), presumably because they have ambiguous meanings. But in this case it's unclear what the goal is: the subject already knows what they are supposed to select, so if they remember, they should just go with what they were told ('groom me' below, as I recall).

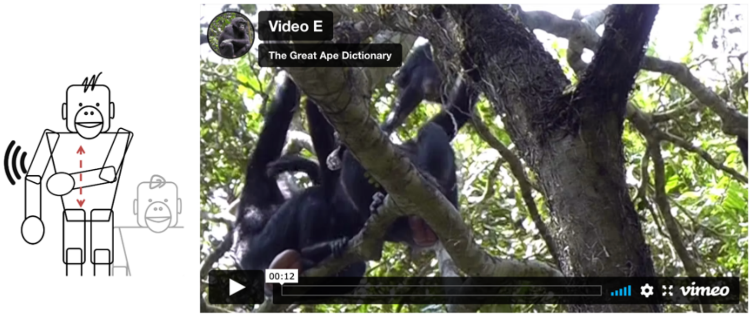

Let's be friendly | Move into a new position | Move away from me | Groom me

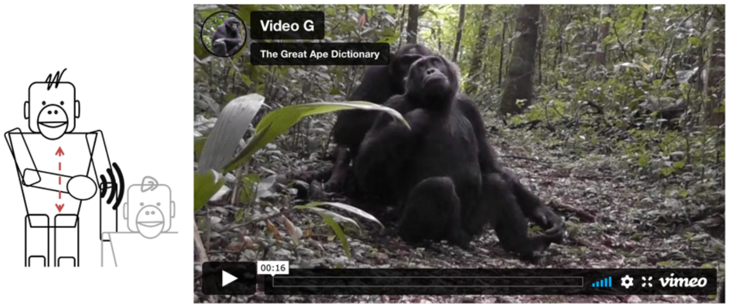

Climb on my back | Groom me | Let's be friendly | Move closer to me

Typos etc.

Leiberman => Lieberman (twice: in the text and in the references)

Figure 1: BLS needs to be explained, I think.
