# Supplementary material for: Towards a great ape dictionary: Inexperienced humans understand common nonhuman ape gestures
Source: PLoS Biol. 2023 Jan 24;21(1):e3001939. doi: 10.1371/journal.pbio.3001939 (PMC9873169; doi:10.1371/journal.pbio.3001939)
Supplement: Attachment — Submitted filename: Response to Reviewers.docx [file pbio.3001939.s004.docx]

**Response to Reviewers**

**Reviewer #1:**

We want to thank Reviewer 1 for their thorough review of our paper. It’s a joy to have one’s paper read so carefully, and their recommendations have undoubtedly strengthened our paper.

*Interpretation of the results*

*The authors write the following in the abstract and in the body of the paper:*

*Abstract: “We show that humans* ***retain*** *an understanding of ape gestural communication, across gesture types and gesture meanings, with information on communicative context providing only a marginal improvement in success. By assessing comprehension, rather than production, we accessed the great ape gestural repertoire for the first time in adult humans. Cognitive access to an ancestral system of gesture appears to have been retained after our divergence from other apes, drawing deep evolutionary continuity between their communication and our own.”*

*Text: “Participants were substantially above chance at assigning the “correct” meanings to chimpanzee and bonobo gestures across types, suggesting that humans* ***have retained their understanding of core features of a gestural system present in our last common ancestor with the Pan genus 6-7 million years ago*** *(Young et al. 2015).”*

We have now tempered these boldfaced sections to reflect that this is just one interpretation (“may retain”, “may have retained”)

*In my view, the boldfaced parts are not justified. The authors show that adult humans have an ability to guess the correct use of some ape gestures. This does not tell us whether they do this (i) by way of general intelligence, which might allow them to guess just as well the use of communicative gestures in fish or birds, or (ii) by way of a dedicated cognitive system that has been retained since our last common ancestor with the Pan genus.*

*For lack of a control (involving for instance bird or fish gestures), I find it impossible to adjudicate between (i) and (ii). Furthermore, when looking at the authors' online game, several things seem striking to me:*

*1. Introspectively, one has a clear sense of guessing, and of NOT understanding the meaning of the gestures. Of course this could conceal unconscious knowledge of a preserved cognitive system. But an alternative is that one is doing one's best with one's general intelligence.*

Yes, we had attempted to lay out various interpretations for how participants may be interpreting these gestures in the paragraph starting line 196, but had not explicitly included general intelligence. We’ve now added to line 200-204 so that it reads: “share an underlying ability to produce and interpret naturally meaningful signals that are mutually understandable because of general intelligence and shared body plans and social goals, or the resemblance of gestures to the actions that they aim to elicit. Despite the importance of context in the interpretation of human communication…”

*In this respect, it might be interesting to comment on the subjective confidence that subjects assigned to their judgments, although here too the data would need to be compared to a control of some sort (e.g. one's subjective confidence in understanding human gestures.) Did the subjects get an impression that they were guessing, or did they think they understood a communicative gesture?*

Unfortunately, we do not have a baseline for participant confidence to compare to, so our findings on confidence must be interpreted with caution. However, we were very interested to address this point and have conducted a small additional analysis to do so. Our hypothesis for participant confidence was that participants should be more confident for gestures with just one main meaning compared to gestures with multiple meanings. We do find a significant effect, but it is not large especially considering the size of our dataset, so we do not want to overinterpret it. We have now included this in our results (Line 155): “Participants showed a small but significant increase in confidence for gestures with a single correct meaning (mean=6.05, SD=2.16), than for Ambiguous gestures (mean=5.88, SD=2.31; t-test: t(81,512)=-166, p=0.049).)

*2. There are cases in which one can guess the meaning of gestures based on general intelligence alone. Examples: -One of the video+drawings figures genitals prominently. This makes a 'let's have sex' meaning plausible.*

*-Similarly, the video of the mouth stroke seems to involve an ape chewing, which makes the correct meaning ('give me that food') particularly plausible.*

We agree that while we tried to cut as close before and after the gesture action as possible, some contextual information still overlaps and features prominently in certain videos. We have added (Line 207): “However, there were some gesture types for which we could not completely remove contextual information because it overlapped with gesture production, for example the presence of food in some Mouth Stroke gestures.”

-*Some videos involve iconic gestures, such as some of the grooming gestures.*

We agree, resemblance to intended outcomes may also be a mechanism used by other great apes to deploy/interpret these gestures. We have now included this more explicitly (Line 202): “…goals, or the resemblance of gestures to the actions that they aim to elicit.”

*As things stand, the authors show that there is some understanding of ape gestures in human adults; they do not show that this is because of a preserved cognitive system rather than because of general intelligence.*

We agree, and hope that the new changes more explicitly reflect our caution about the interpretation of the underlying mechanism.

Selection of the target gestures

1. More should be said about the criteria by which the gestures were selected. The article says:

"We selected 10 gesture types for which we were previously able to confirm meaning in both chimpanzees and bonobos (Graham et al. 2018)."

The worry is this: In principle, one would like to test a random sample of the gestures to avoid a selection bias. If the researchers selected those gestures that they thought are most transparent to humans, the result would just be that adult humans understand SOME (appropriately selected!) ape gestures. If the selection is random, one can hopefully infer that adult humans GENERALLY understand ape gestures. There is a sizable difference between the two conclusions, but it's hard to tell from the paper which situation we are in.

We agree that a random sample across gesture types or testing the whole gestural repertoire would be ideal, and we would love in due time to conduct that follow-up study! However, because we need substantial datasets for both chimpanzees and bonobos on the same gesture type used on successful occasions by multiple individuals to establish the gesture type’s meaning(s), the set of testable gesture types available at the current time is considerably smaller. Here we used the 10 most common gesture types with established meanings for both chimpanzees and bonobos and have now included this limitation properly in the paper (Line 106): “We selected the 10 most common gesture types…”

2. A more minor question is this: in principle, one would expect that those gestures that are found with a constant meaning in gorillas, chimpanzees and bonobos are found with the same meanings in humans as well. This is so because gestures that are shared among the three species should presumably be found in their most common ancestor and could presumably be preserved in humans. Gestures that are not shared between bonobos/chimpanzees and gorillas might be an innovation (whether in form or in meaning) of the bonobo/chimpanzee lineage, and not shared with humans. I assume it is for practical reasons that the authors focused on bonobo/chimpanzee gestures, but here too additional explanations would help.

Yes! Testing understanding of gorilla or orangutan gestures would be great! As the reviewer points out as well as being our closest relative, we are also chimpanzee and bonobos closest relative – closer than gorillas or orangutans. We do know that a large proportion of gesture forms are shared across chimpanzees, bonobos, and gorillas, and a smaller but substantial number across all non-human apes. However, unfortunately, gesture meanings are not yet established for gorillas or orangutans, although our research group is working hard to correct that. We have now explained this more clearly (Line 108): “Chimpanzees and bonobos are humans’ closest living relatives (we are also theirs, with the split from humans more recent than the last common ancestor shared between *Pan* and *Gorilla*; Hobolth et al. 2007). While in principle, given the overlap in gesture repertoires across all apes (Byrne et al. 2017), we would predict that gorilla and orangutan gestures may also be salient to humans, the meanings for gestures in these ape species are not yet established.”

3. Similarly, it might be worth explaining in greater detail what is expected in humans when bonobos and chimpanzees assign a different meaning to the same gesture, as is the case for 'directed push' below, from the Supplementary Materials.

We don’t have specific predictions for humans choosing the meaning associated with either of the species, just that they should choose the appropriate meaning for the communication event. Contextual information within the video may supplement this understanding. We have added (Line 127): “Some of the gesture types, e.g. Directed Push, have different primary and alternate meanings, e.g. “Climb on my back” for bonobos and “Move to a new position” for chimpanzees. For these, as for ambiguous gestures, we expect participants to answer with the correct response for the specific video.”

*Title: The title of the contribution isn't optimal. First, it's a stretch to call a 10-gesture list of 'dictionary'. Second, 'language-using' is almost pleonastic when applied to humans, and it doesn't bring any useful information. Third, and less importantly, I find the generic claim a bit overblown ('understand non-human ape gestures'): true, to some extent, on an existential reading ('some non-human ape gestures'); whether it's true more generally (on a generic readings) remains to be seen (this depends in part on clarifications requested above about principles of gesture selection).*

Thank you for your suggestions. We have changed the title to reflect that this is part of a process that aims to build a type of dictionary, and that removes our animal communication contortion around language use. The new title reads: “Towards a great ape dictionary: naïve humans understand non-human ape gestures”

*Meaning: The term 'meaning' is used in the paper for 'Apparently Satisfactory Outcomes' (ASOs). This should be clarified in the paper. To anyone familiar with linguistics, it seems entirely implausible that ASOs are meanings, i.e. the cognitive representations of the informational content of gestures. Rather, ASOs are convenient ways to operationalize the various uses of gestures, but it seems clear that the core meanings of these gestures haven't been discovered yet. This is not a problem for the present study, but some readers will be misled if the authors do not state clearly what they understand by 'meaning' (namely ASOs).*

Thank you, that’s an important distinction that we have now clarified on Line 106 “We selected the 10 most common gesture types for which we were previously able to confirm “meaning” in both chimpanzees and bonobos, determined by recipient responses that consistently satisfy the signaller (Graham et al. 2018)”

*Details*

*–It is fantastic that the stimuli are open access. It would be even better if the relevant gesture drawings and videos were linked to the relevant parts of the paper, especially around Figure 1. This way readers could see exactly what subjects had to assess.*

Thank you, we’re hopeful these will be helpful for other researchers. We’ve now added links to the materials and video examples where-ever they’re first mentioned (e.g. Lines 95 o 102) to hopefully make this easier to interact with. We have also added a link to the gesture drawings and video examples in the legend for Figure 1.

*–I thought several of the videos were very hard to decipher, in which case the drawings played an essential role. It might be worth commenting on the fact that subjects saw a *combination* of drawings and videos. In particular, the drawings clearly tell the viewers which part to focus on (as it appears in red).*

Yes, that is very important, and was hidden in the methods section. We have now added much earlier (Line 101): “Each video was accompanied by a simple illustration of the gesture to assist naïve viewers in identifying the gesture action (<https://greatapedictionary.ac.uk/gesture-videos2/)>.”

*–When using https://app.gorilla.sc/openmaterials/344409, I got an error message when clicking on: Great ape dictionary: Error creating schedules*

Thank you for catching this, it was an error in the naming of one of the linked files, which we have now fixed.

*–In the version of the experiment for Kids under 12, subjects are told the right answer after they have submitted theirs. But some gesture videos appear twice (example below), presumably because they have ambiguous meanings. But in this case it's unclear what the goal is: the subject already knows what they are supposed to select, so if they remember, they should just go with what they were told ('groom me' below, as I recall).*

We don’t use any of the data from the Kids under 12 version – that was a game for outreach, and to make it more immediately fun we chose to let them know how they were doing along the way. We duplicated some videos in this gamified version because we removed videos with age-inappropriate sexual meanings.

*Typos etc.*

*Leiberman => Lieberman (twice: in the text and in the references)*

Thank you for catching that, we have fixed this error.

Figure 1: BLS needs to be explained, I think.

We have now added an asterisk next to BLS in Figure 1 and added “*BLS = Big Loud Scratch” to the caption (as editing in the full text in the figure would make the text along the x-axis too long and distort the figure).

**Reviewer #2:**

*This was a very nice and interesting study reporting a novel citizen science experiment that examined people's comprehension of great ape (chimpanzee and bonobo) gestures. It was well written and well structured. The study used a simple but elegant design of presenting members of the public with videos of great ape gestures whose meanings were known, which they had to judge the meaning of in a forced-choice design. Participants were better than chance at judging the correct meaning of ape gestures, and interestingly context made only a relatively small difference.  The  authors are to be congratulated on the very impressive sample size: managing to engage over 17 000 particpants, of which 5600 provided useable data is a great achievement.  Although assessing human perception in detecting animal signals is not itself new, this citizen science study is the first of its kind, including to explore it on such a large scale. The public engagement for the study is a great achievement.* 

Thank you for your enthusiasm about this study!

*The study was well designed, well executed. Nevertheless, there are some aspects that warrant attention, particularly the framing, rationale as well as the interpretation.  There are also a few omissions in the analyses which need attention (detailed below).

A key premise of the paper is that it is a conundrum that human gestures do not map onto great ape gestures (except in infants) (L65). There are two issues with this stance, First, the statement itself seems evolutionarily counter-intuitive, given evidence of extensive overlaps among other great ape gesture repertoires (making it most likely that humans overlap too), and there is also evidence of considerable continuities in the production and perception of communicative expressions of humans and other great apes in other modalities too (facial expressions and vocalisations).  In this sense, it  should be expected that humans can perceive gestures of apes too, above chance. This doesnt undermine their result, it just means its consistent with what one would expect. however, the statement that he producton of human gestures don't map onto ape gestures (at least from what is reported here) appears to be based on an absence of evidence, rather than evidence of absence. There is no study reported in the paper which addresses this, its only implied in various statements. Clarifying this is important as otherwise 'the conundrum' could more be just a case that production hasn't yet been adequately investigated. A study focussing on production would be a very exciting complement to this study.*

Thank you, we completely agree and have now clarified that point (Line 62-71):

“Great ape repertoires show substantial overlap across species, including overlap among ape species more distantly related than chimpanzees, bonobos, and humans (Byrne et al. 2017, Genty et al. 2009, Graham et al. 2017, Hobaiter & Byrne 2011, Schneider et al. 2011). As a result, we would expect humans to retain the use of this system of ape gestural communication; but, to date, the use of naturalistic ape gestures appeared to be absent in human communication. Humans are highly gestural, deploying deictic, iconic, conventional, co-speech/co-sign, among other kinds of gestures. However, this itself is part of what makes studying gestural overlap between adult humans and other apes challenging. Gestures shared with other apes may be masked by the myriad ways that people signal with their hands and body.”

And (Line 75): “Unpicking gestures from the great ape repertoire in naturalistic adult human gesturing may not be impossible, but it will take a substantial collaborative effort to one day do so. In the meantime, there are other methods at our disposal.”

*Also regarding framing, the paper starts out by resting on rather a strawman argument regarding a discontinuity between language and non-human communication (L30). As the authors themselves will be aware, evidence is revealing greater continuities than previously assumed.  The authors contrast ape gestures to the apparently 'fixed' nature of primate alarm calls (L34-36); however there are multiple studies that now challenge this, for instance, studies of chimpanzee and orangutan alarm calls showing that caller's flexibly adjust their signals according to  audience presence and knowledge (also potentially in other primates too e.g. audience effects in Thomas langur calls). Likewise, the next paragraph (L42) states that, by contast to animal signals,  language 'does more than broadcast information'. Yet we now know that many animal signals also do more than broadcast information, as well as non-linguistic signals in humans. This part would warrant adjustment to avoid undermining progress that has recently been made to move away from such argumentation.*

We agree fully with the reviewer! But have also experienced continued scepticism about arguments for continuity outside of the comparative/animal communication community, where the case for language as qualitatively difference, special, and separate continues – thus, we probably oversold a little here. We’ve adjusted this phrasing to provide a better balance. We’ve now added to Line 30: “However, more and more research has started to unveil language’s deep phylogenetic roots: from the way other species combine signals to change the meaning of an utterance (Engesser & Townsend 2019); to their use of social inference in communication (Warren & Call 2021); to how behavioural and social contexts seem to disambiguate signal meanings (Graham et al. 2020).”

*The analogy made in L37-41 seems a little over-simplistic.  Even in the absence of an audience, a signal may still be produced with some intention or be based upon a context which ordnarily would have a communicative goal. Linguistic signals also can also be used in this way, not just nonlingustic ones. E.g. When picking up a hot pan alone, one might still say  "that was really hot!, That hurt.' - this may contain intentionality even in the absence of an audience. Moreover, there is also evidence that signalling to oneself may also serve other cognitive functions for the signaller. A signal even in the absence of an immediate audience may still have evolved to be communicative, even in the absence of an audience at that given moment. There is a lot to unpack here* 

We agree – there’s definitely a lot to unpack here. For example, the ability to talk to ‘oneself’ and to what extent this might imply intentional use. We’re not sure that it would be possible to unpack this fully here, but we have added to the example to try to better convey the complexity of what might be involved in doing so (Line 45): “We can use it in the absence of the stimuli that we were originally responding to. We would stop using it once our recipient indicated that they understood. We might even use it to talk to ourselves.”

*Methodology: The experimenters collected data on age, gender and experience with primates yet these variables didn't seem to be included in the analyses? It would be interesting and worthwhile to examine age and experience effects in particular, especially as the authors allude that perhaps human gestures get less 'ape-like' with age?  It would also be interesting to see the demographic information about the sample population, was this collected?*

While we agree there are interesting questions around participant identity and understanding, we are concerned it may be beyond the scope of this paper. We had no specific hypotheses about gender, and so did not test that, and the other variables were used to exclude participants. While age in particular could be of interest, our data set can not be used to address this as we would need data from a much younger demographic e.g. children in whom language skills are developing, rather than adults in whom it is fully expressed. As children under 12 years old are not able to provide informed consent or assent in an online environment, their data were not retained.

We have added to the methods (Line 237): “Demographic data was used to exclude participants who were too young to consent; who had experience with non-human primates; and who had done the experiment before.” (These points are also specified in the Data Exclusions section)

*Minor comments:

L33: One of the references used to support a sentence on primate alarm calls is actually about meerkats (Manser (2001)*

Thank you for catching that, we meant to just write “alarm calls” and have now edited (Line 35)

*L156: There is also evidence that humans can perceive the emotional content of primate calls, so this finding is not just limited to gestures.*

We are excited to be able to reference this new work and have now cited the relevant preprint (Line 193): “That our participants were able to interpret primate signals complements recent findings that suggest humans may be able to perceive affective cues in primate vocalizations (Debracque et al. 2022, preprint).”

*L236: For gestures with multiple meanings, there is a bit of a bias introduced in the experimental design which could increase chances to get at least one right if the 'second meaning' was always added as one of the options. To ensure chances remain at 0.25 per trial, it would have been good to have done a counterbalanced design where only one meaning option (first or second) was available. It's possible this was somehow controlled for and I missed it.* 

For all gestures we ran the binomial for the primary meaning with chance at 0.25 (now clarified on Line 139 (“significantly higher than expected by chance (0.25)”) and in S1 Table. Then for ambiguous gesture types we ran a binomial for the three remaining meanings once the primary was removed, making chance 0.33 (now clarified in S1 Table).

*Figures: The figures are nicely presented, just the fonts could be adjusted to be more legible*

Thank you, we have decided to move Figure 2 to Supplementary Materials to reflect your and Reviewer 3’s views and have adjusted the font to improve legibility.

*Table 1: Wasn't clear what the third column in the table was (unlabelled)*

Thank you, we have now fixed the table labels.


**Reviewer #3:
[identifies herself as Heidi Lyn]**
 *This article is an extremely interesting and modernized take on an older method of studying linguistic competence, comprehension. The authors present ape gestural data online and human participants indicate their understanding of the meaning of the gestures, to great success. I think the study is acceptable, with just a few clarifications to improve readibility, mostly.*

Thank you for your enthusiasm and your helpful suggestions.

*Lines 92 - 96 - some examples of gestures with single and multiple meanings, as well as examples of primary and secondary meanings would be helpful. Also a description of how those meanings were determined.*

We agree that this needed further clarification and have substantially expanded the two paragraphs starting on Line 106 (begins “We selected the 10 most common gesture types…”) to address these three points.

*Line 102 - why is the completion rate so low? Is it possible there is some bias introduced here?*

That is a very good question – we believe it is low because the experiment took some time to complete (~ 15 min) and was unpaid. Completion rates of under 30% are not unusual in online studies, and our completion rate for participants not excluded for other reasons was 33%. We have now added the number of participants excluded at each stage in the Participant exclusions section to provide more transparency on who was excluded for which reason (Line 244).

Line 107 - are these binomials based on a 50% chance rate? But the participants chose from 4 possible answers? It would be helpful to clarify that chance would actually be much lower than 50% in the results, otherwise the percentage correct does not look impressive - because your numbers of trials are so high, a marginal improvement over chance would still be significant. Have you considered analyzing individuals' mean scores with a one-way t-test? Then you could also report on whether individual participants were able to recognize the entire repertoire.

They are based on a 25% success rate, which we have now clarified on Line 139: “…significantly higher than expected by chance (0.25)” and in S1 Table.

*Page 6 - I find both Figure 2 and Table 2 hard to interpret, although Figure 2 has me more puzzled. Is each gesture being added to the glmm as a separate predictor(Fixed effect)? Or a moderating variable? And you state there are 10 gestures, but only 7 meanings in the table? And their estimates in the glmm seem to vary widely from negative to positive. I'm not sure these visuals aid in the understanding of your findings or distract from them as the reader tries to interpret.*

Thank you, we agree that this figure does not clearly and quickly convey the core finding of our study and have now moved it to the Supplemental Materials to avoid confusion. We have clarified the text and expanded the legend to aid in its interpretation.

*Line 133 - this is a really interesting finding that I think should be emphasized more. When there was an alternate meaning, participants averaged closer to 75% choosing one of the possible meanings. In other words, choosing a completely unrelated meaning was much less likely than the initial percentages correct may suggest.*

Thank you – (outside of Object shake) the primary meaning was much more successfully detected than an alternate meaning; however, as you say, the alternate meaning was often still somewhat more successfully detected than the incorrect meanings, and together success rates were typically above 75%. We have now added to the discussion to better highlight this (Line 189): “This ability was present across both the functionally more fixed and the flexible gestures that are deployed with more than one meaning. Participants were highly successful at detecting the meaning for which gestures were used in the specific instance of communication that they saw. Where gestures had alternate meanings, these were also detected more often than chance in two gesture types.”

*Discussion and Introduction: It would be great if there were some mention of the long history of comprehension as an earlier/better measure for language or symbolic competence in many species: e.g. 
Children: Shipley, E. F., Smith, C. S., & Gleitman, L. R. (1969). A Study in the Acquisition of Language: Free Responses to Commands. Language, 45(2), 322-342.* [*https://doi.org/10.2307/411663)*](https://doi.org/10.2307/411663))*, 
marine mammals: Herman, L. M., Richards, D. G., & Wolz, J. P. (1984). Comprehension of sentences by bottlenosed dolphins. Cognition, 16(2), 129-219.* [*https://doi.org/10.1016/0010-0277(84)90003-9*](https://doi.org/10.1016/0010-0277(84)90003-9) *and apes: Sevcik, R. A., & Savage-Rumbaugh, E. S. (1994). Language comprehension and use by great apes. Language & Communication, 14(1), 37-58.*

Thanks so much, this was not an angle we had considered, but is obviously important and relevant to our approach! We have added (Line 87): “This type of comprehension study has historically been employed to test nonhuman species on comprehension of human language (Herman et al. 1984, Sevcik & Savage-Rumbaugh 1994), but here we flip the paradigm to test humans on nonhuman communication.”

*An Extended Data Table is mentioned several times, but I don't see it?*

We included this in our submission, so hope that it was just an error in what you were given access to. We have resubmitted it this time to be certain. Additionally, all our data and code are available in an open access repository at: https://github.com/Wild-Minds/GreatApeDictionary
