# Supplementary material for: Towards a great ape dictionary: Inexperienced humans understand common nonhuman ape gestures
Source: PLoS Biol. 2023 Jan 24;21(1):e3001939. doi: 10.1371/journal.pbio.3001939 (PMC9873169; doi:10.1371/journal.pbio.3001939)
Supplement: Attachment — Submitted filename: PLOSbio_ResponseToReviewers.docx [file pbio.3001939.s005.docx]

Reviewer #1:

*I am happy to recommend publication of this very, very interesting paper (kudos to the authors for paying great attention to the objections), but I would suggest that some additional clarifications/improvements be made.

1. Optionally: ***this should be left to the authors***, but personally I would prefer a title that's less generic, e.g. with 'some':  e.g. "Towards a great ape dictionary: naïve humans understand some non-human ape gestures."
The generic suggests that naïve humans *generally* understand ape gestures, and this seems to me to be an overstatement. Something less generic would be more accurate given the small number of gestures investigated. I do understand that this might take away from the impact of the article, so I'll understand if the authors prefer something that makes a bigger splash (although in the long term, I am not sure this is the best strategy).*

Thank you, we agree that more specificity is important, and so have opted to add the word “common” to the title to reflect that it’s not just some of the gestures but the most common great ape gestures that were selected from the repertoire.

*2. I would recommend that the abstract be rewritten to state clearly and explicitly that humans understand (some) ape gestures, but that this could be for 2 reasons: 1. an innate communication system has been retained. 2. general intelligence allows humans to guess the meaning of some ape gestures. Again, this might weaken the short-term impact of the paper, but it's a far more accurate assessment of the finding and will help with the long-term credibility of this (wonderful) line of research.*

Yes, we would also rather have a clear and accurate paper than a sensational one! We have added the caveat to line 19/20: “We show that humans may retain an understanding of ape gestural communication (either directly inherited or part of more general cognition)…”

*3. Still in the abstract, I think the authors could say in so many words that the 10 gestures studied were the most common ones. This strengthens the authors' point, as this is a clear criterion, which might defuse the reader's worry that the choice of items was biased.*

Very important point - we’ve clarified more in the abstract now adding “10 of the most frequently used ape gestures” and “we accessed part of the great ape gestural repertoire”.

*4. The final discussion could be clarified. The following is a great improvement:

"It remains unknown whether the great ape repertoire itself is biologically inherited (Byrne et al. 2017), or whether apes - now including humans - share an underlying ability to produce and interpret naturally meaningful signals that are mutually understandable because of general intelligence and shared body plans and social goals, or the resemblance of gestures to the actions that they aim to elicit"

But I think this is still insufficiently accurate. This reads as if there are 2 salient theoretical possibilities.

Possibility 1: In apes and humans alike, the ape repertoire is (genetically) inherited.
Possibility 2: In apes and humans alike, the ape repertoire is understandable because of general intelligence etc.

But this incorrectly suggests that the most salient theories are ones in which apes and humans share an ability in this connection. This is not correct, as a very salient possibility is a third one, namely:

Possibility 3: In apes, the ape repertoire is (genetically) inherited. In humans, the ape repertoire is understood through general intelligence etc.

Possibility 3 is a deflationary account of the authors' finding. It's useful to mention it very clearly because it might trigger further research to prove or disprove the point.*

Thank you, we did not intend to suggest that there were only two explanations (or indeed only three) and so have added a sentence to encourage caution in this interpretation (Line 209): “These are not the only possible explanations, e.g. gestures could be biologically inherited in nonhuman apes but understood by humans through other cognitive mechanisms, and we need to continue to develop innovative methods such as these video playbacks to address remaining unknowns.”

*5. The term 'meaning'

I appreciate the authors' response to my earlier suggestion, copied at the bottom of this report. But I think it would be easier if they stated clearly and explicitly at the beginning of the paper something along the following lines:

In this article, we use the term 'meaning' to refer to a reaction type, specifically to what has been called an 'apparently satisfactory outcome' (ASO) in the earlier literature.

with appropriate references to the literature, and examples. As I wrote earlier, without such a definition, linguists will be baffled by this use of the term 'meaning'. And it just takes a few clear words to provide a clear definition.*

We have added to Line 34 “(we use “meaning” in this article to refer to signal functions and Apparently Satisfactory Outcomes; Hobaiter & Byrne 2014, Smith 1965)”

*6. Human playback experiment
The description copied below (a version of which appears in the discussion) has the advantage of coolness ('performing ape-like playback experiments with humans, what a neat idea!').  But I think it doesn't do justice to the experimental situation. First, the authors employ a standard methodology of psychology/psycholinguistics: the subjects are just asked to guess the meaning of various things in perception. Second, no playback experiment with animals would look anything like this, as the human subjects are asked to do something entirely unnatural (they need to read complex instructions and provide guesses about meanings/functions).  I would recommend restating all this far more simply. The coolness factor might decrease, but credibility would increase.

"We employ a method regularly used in studies of non-human primate communication, a 'play-back' experiment, in which recipient behaviour is analysed following exposure to a signal (Fischer et al. 2013, Radick 2005). This type of comprehension study has historically been employed to test nonhuman species on comprehension of human language (Herman et al. 1984, Sevcik & Savage-Rumbaugh 1994), but here we flip the paradigm to test humans on nonhuman communication. While language-competent humans seem to no longer typically produce"*

Thank you – the parallel to nonhuman ape playbacks was a suggestion by another reviewer, and so we have now tried to adapt it to suit both views. We have added to line 93: “Of course, our experimental paradigm is more conventional in the human psychology literature, and has the advantages that, with humans rather than nonhumans, we are able to conduct tests with untrained participants and to use text responses in this match-to-sample type paradigm.”

*As before, I think this is a great experiment and a super interesting paper!*

Thank you so much, we are glad you enjoyed it!

*EARLIER SUGGESTION ABOUT 'meaning'

Meaning: The term 'meaning' is used in the paper for 'Apparently Satisfactory Outcomes' (ASOs). This should be clarified in the paper. To anyone familiar with linguistics, it seems entirely implausible that ASOs are meanings, i.e. the cognitive representations of the informational content of gestures. Rather, ASOs are convenient ways to operationalize the various uses of gestures, but it seems clear that the core meanings of these gestures haven't been discovered yet. This is not a problem for the present study, but some readers will be misled if the authors do not state clearly what they understand by 'meaning' (namely ASOs).

Thank you, that's an important distinction that we have now clarified on Line 106 "We selected the 10 most common gesture types for which we were previously able to confirm "meaning" in both chimpanzees and bonobos, determined by recipient responses that consistently satisfy the signaller (Graham et al. 2018)"*

We believe this was a suggestion that we addressed and that is for our reference in Point 5? We have addressed this now on line 34 “(we use “meaning” in this article to refer to signal functions and Apparently Satisfactory Outcomes; Hobaiter & Byrne 2014, Smith 1965)”
